# Generation of an Attenuated Tiantan Vaccinia Virus Strain by Deletion of Multiple Genes

**DOI:** 10.3389/fcimb.2017.00462

**Published:** 2017-10-31

**Authors:** Yiquan Li, Yilong Zhu, Shuang Chen, Wenjie Li, Xunzhe Yin, Shanzhi Li, Pengpeng Xiao, Jicheng Han, Xiao Li, Lili Sun, Ningyi Jin

**Affiliations:** ^1^Medical College, Yanbian University, Yanji, China; ^2^Institute of Military Veterinary Medicine, Academy of Military Medical Science, Changchun, China; ^3^Changchun University of Traditional Chinese Medicine, Changchun, China; ^4^Institute of Virology, Wenzhou University Town, Wenzhou, China; ^5^Department of Head and Neck Surgery, Tumor Hospital of Jilin Province, Changchun, China

**Keywords:** attenuated vaccinia virus vector, homologous recombination, vaccinia Tiantan strain virus, virulence, immunogenicity

## Abstract

An attenuated vaccinia virus-MVTT_EAB_-was constructed by deletion of non-essential gene segments related to the immunomodulatory and virulence functions of the vaccinia virus Tiantan strain (VVTT). The shuttle plasmids pTC-EGFP, pTE-EGFP, pTA35-EGFP, pTB-EGFP, and pTA66-EGFP were constructed and combined with the early and late strong promoter pE/L and EGFP as an exogenous selectable marker. Then, through the homologous recombination technology and Cre/loxP system, the following gene fragments were gradually knocked out one by one: TC7L-TK2L, TE3L, TA35R, TB13R, and TA66R. Ultimately, the five-segment-deleted attenuated strain MVTT_EAB_ was obtained. Knockout of these segments and genetic stability of MVTT_EAB_ were confirmed, and it was also shown that knockout of these segments did not affect the replication ability of the virus. Further, a series of *in vivo* and *in vitro* experiments demonstrated that the virulence of MVTT_EAB_ was attenuated significantly, but at same time, high immunogenicity was maintained. These results indicate that MVTT_EAB_ has potential for clinical use as a safe viral vector or vaccine with good attenuation and immunogenicity.

## Introduction

The vaccinia virus (VV), which is used as a smallpox vaccine, belongs to the family Poxviridae and genus Orthopoxvirus. It is a complex double-stranded DNA virus that replicates and forms peculiar viral particles in host cells. The VV genome size is 185–200 kb, and it can encode about 200 different proteins (Qin et al., 2015). The highly conserved central part of the genome, which comprises the majority of the VV genome, contains the essential genes that play a key role in virus replication, such as transcription, DNA replication, and viral particle assembly. In contrast, the genes present at both ends of the genome are commonly used to identify species or host specificities and to encode proteins that regulate the host immune system and virulence factors (Liu and McFadden, 2015). Genome analysis of VV has led to new breakthroughs in the phylogeny and evolution of VV, and has shown that the VV proteins are more analogous to eukaryotic proteins than bacterial proteins. Research findings have indicated that the genes in this virus are likely to have come from their eukaryotic host genes via horizontal gene transfer, and that these slow and sustained processes have contributed to the evolution of VV. Many laboratories have initiated research on the use of naturally evolved strains for vaccination, especially research on improving the safety of VV and other poxviruses. In particular, many studies have shown that VV is useful for the study of vaccine vectors and exogenous gene expression systems (Garcia-Arriaza et al., 2013; Noisumdaeng et al., 2013; Adelfinger et al., 2014; Remy-Ziller et al., 2014).

VV is widely used in the field of gene engineering vaccine vectors and exogenous gene expression systems (Garcia-Arriaza et al., 2013; Adelfinger et al., 2014). Furthermore, live genetically engineered vector vaccines using VV as the vectors have been developed, and to be directed against more than 30 species of virus including herpes simplex virus, hepatitis A virus, hepatitis B virus, human immunodeficiency virus, and others, such as the genetically engineered vaccine of rabies virus glycoprotein (G) expressed using VV Copenhagen strain as the vector (Pastoret and Brochier, 1996), that of Newcastle Disease Virus fusion protein (Fusion protein, F) expressed using VV Elstree strain as the vector (Meulemans et al., 1988), that of vesicular stomatitis virus G protein expressed using VV vaccine Western Reserve strain as the vector (Mackett et al., 1985)and etc. Although research on the use of VV as vectors has made remarkable progress, there are still some limitations, such as difficulty in the screening of recombinant viruses and the insertion of exogenous selection markers, and the complexity of vector construction procedures (MacNeil et al., 2009). Currently, reducing the side effects of VV vaccines, improving the efficiency of vector vaccines, and simplifying the preparation procedures are hot topics in research on VV vectors.

In this study, the Cre-loxP recombination system was used to delete the following non-essential gene segments of the VVTT strain one by one to ultimately to obtain the attenuated strain MVTT_EAB_: TC7L-TK2L (15,262–25,450: TC, TC, TC, TC, TC, TC, TC, TN, TN, TM, TM, TK1L, and TK), TE3L (47,348–47,921), TA35R (138,881–139,570), TB13R (173,213–174,206), and TA66R (161,870–162,817). The knockout fragments included a variety of virulence-related genes, host-related genes and immunomodulatory genes. The TC7L-TK2L fragment is involved in the regulation of pathogenicity, virulence, and host range of the gene. NYVAC, as one of the most successful gene-knockout attenuated VV vectors, was obtained by phenotypic attenuation after knockout of the C-K1L fragment (12 ORFs) (Tartaglia et al., 1992). E3L is a virulence and immunomodulatory gene that encodes a protein which inhibits the activation of interferon-induced pathways, thereby inhibiting the antiviral response of host cells (Guerra et al., 2011). Several previous reports have shown that knockout of E3L in the Copenhagen, WR and NYCBH strains of VV can result in highly attenuated virus (Vijaysri et al., 2008). A35R is a virulence gene that modulates the adaptive immune response. A study has shown that knockout of A35R can lead to a decrease in viral replication capacity and reduce viral virulence (Brennan et al., 2015). B13R is a non-essential immunomodulatory gene with anti-apoptotic and anti-inflammatory effects and has sequence homology with serpins (Legrand et al., 2005). The TA66R gene encodes a viral hemagglutinin that has similar function to A56R of the VACV WR strain, which is inhibition of cell fusion. The total size of the deleted sequences was about 20 kb, which accounted for 10.6% of the sequence of the VVTT genome. Enhanced green fluorescent protein (EGFP) was used as the exogenous screening marker, and the Cre/loxP system was introduced into the shuttle vector plasmid for knockout of exogenous selection markers. In subsequent *in vitro* and *in vivo* experiments, MVTT_EAB_ was found to have good attenuation and good immunogenicity as a vaccine. Thus, the recombinant VVTT strain MVTT_EAB_ with the five gene segment deletion that was constructed in this study may have a wide range application prospects as a live vector vaccine and exogenous gene expression vector. Further, the number of cycles of recombination for constructing recombinant VVs could be significantly reduced and the efficiency of screening could be significantly improved by using the construction and screening strategies used in the present study. Thus, these construction and screening methods for the recombinant virus could present optimized solutions for studying new recombinant VV vector vaccines.

## Materials and methods

### Cells, viruses, and animals

BHK-21, HeLa, PK-15, MDCK, and Vero cells were purchased from the China Center for Type Culture Collection. All cells were cultured in Dulbecco's modified Eagle medium (DMEM) (Invitrogen, Beijing, China) supplemented with 10% fetal bovine serum (FBS) (Hyclone, Beijing, China), 1% streptomycin (10 mg/mL), and 1% penicillin (10,000 U/mL). The VVTT strain (GenBank accession no. AF095689) was obtained from the Institute of Virology at the Chinese Center for Disease Control and Prevention.

Female New Zealand white rabbits and female BALB/c mice (aged 3–8 weeks) were obtained from the Experimental Animal Center of the Academy of Military Medical Sciences of China.

The animal experimental protocols were approved by the Institutional Animal Care and Use Committee of the Chinese Academy of Military Medical Science, Changchun, China (10ZDGG007). All surgical procedures were performed under sodium pentobarbital-induced anesthesia, and all efforts were made to minimize suffering.

### Construction of VVTT transfer vectors

We constructed the pSK-TC-EGFP, pSK-TE-EGFP, pSK-TA35-EGFP, pSK-TB-EGFP, and pSK-TA66-EGFP vectors with standard gene synthesis techniques (Kan et al., 2012). The pSK-TC-EGFP vector has a DNA fragment containing TCL-loxP-PE/L-EGFP-loxP-TCR sites, and both ends of the EGFP fragment had *EcoR*I and *Pst*I restriction enzyme sites. The other four vectors were constructed using the same strategy. These five vectors were constructed by Shanghai Generay Biotech Co. Ltd. The five transfer vectors were identified by digestion with *EcoR*I and *Pst*I.

### Construction of MVTT_EAB_

**Figure 1:**
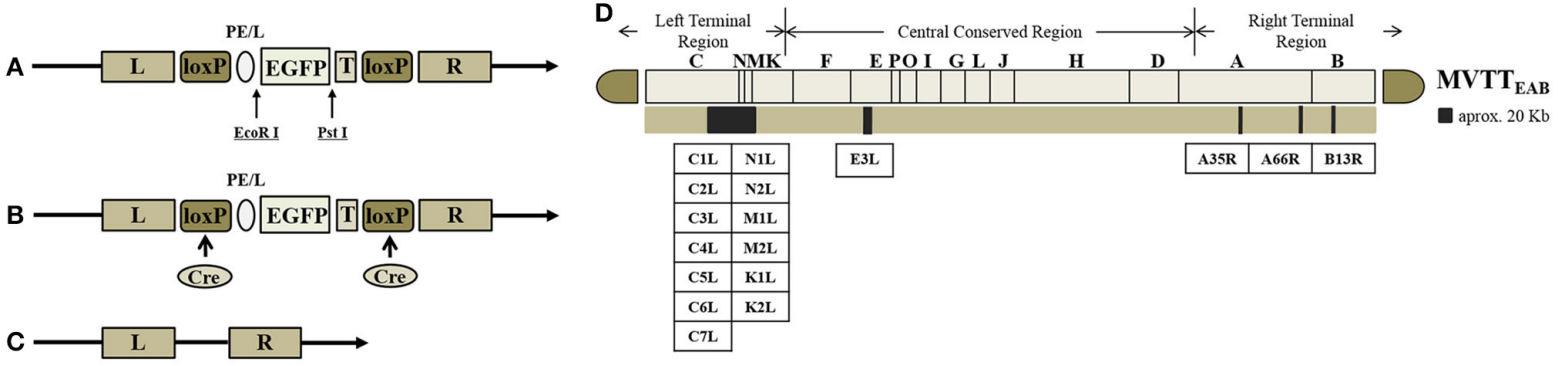
Construction of MVTT_EAB_–an attenuated vaccinia virus with five deleted gene segments. Structure of the plasmid vector **(A)**, recombinant vaccinia virus **(B)**, and gene-deleted virus **(C)**. EGFP as a screening marker was inserted at the TC7L-TK2L gene site of VVTT **(A)** and driven by the synthetic early/late promoter (pE/L) to obtain the recombinant virus with deletion of the TC-TK2L gene but containing the EGFP gene **(B)**. The recombinant virus VVTTC^−^ was generated through Cre/loxP site-specific recombination to remove the EGFP gene **(C)**. The same method was used to successively knock out the other four segments: TE3L, TA35R, TB13R, and TA66R. Ultimately, the five-segment-deleted attenuated strain MVTT_EAB_ was obtained. T, termination signal of vaccinia virus; loxP, loxP site; Cre, Cre recombinase. **(D)** The TC7L-TK2L, TE3L, TA35R, TB13R, and TA66R genomic deletions were found in the viral genome.

The TC, TE, TA35, TB, and TA66 genes were replaced with the EGFP gene to generate the recombinant virus MVTT_EAB_ (Figure 1D). The BHK-21 cells were prepared in six-well plates into which VVTT was added at an MOI of 0.1. The plates were cultured in DMEM containing FBS, streptomycin and penicillin (as mentioned previously) for 2 h before transfection with the mixture of the shuttle plasmid pSK-TC-EGFP and Lipofectamine 2000 (Invitrogen). After culture for 72 h, the recombinant virus was released by three freeze-thaw cycles and used for further infection. Under an inverted fluorescence microscope, green fluorescent plaques were picked out, and this purification process was repeated six times until the monoclonal fluorescent plaque (i.e., the recombinant virus rVVTT-C^−^EGFP^+^) was purified. rVVTT-C^−^EGFP^+^ and the plasmid pVAX1-Cre were co-infected/transfected in BHK-21 cells and cultured for 72 h. Then, the non-green fluorescent plaques (i.e., the non-EGFP-expressing virus) were picked out and purified; this step was repeated six times. Deletion of the TC and EGFP genes in the recombinant virus rVVTT-C^−^ was confirmed by PCR. This process was used to successively delete the other four segments—TE, TA35, TB, and TA66—from the recombinant virus rVVTT-C^−^. Ultimately, an attenuated strain of VVTT with five deleted gene segments was obtained MVTT_EAB_.

MVTT_EAB_ was identified by PCR as follows. The MVTT_EAB_ genome was extracted and used as the template for PCR amplification of the partial fragments TC, TE, TA35, TB, and TA66. The amplification protocol was as follows: 95°C for 5 min; 35 cycles of 95°C for 30 s, 57°C for 30 s, and 72°C for 30 s; and a final extension at 72°C for 10 min. The VVTT genome was used as a control to identify the recombinant virus MVTT_EAB_. The identity of the products was confirmed by nucleotide sequencing. The sequences of the primers used for identification are shown in Table 1.

**Table 1:** Sequences of identification primers and corresponding Tm values.

**Primers**	**Sequences (5′-3′)**	**Fragment (bp)**	**Tm (°C)**
F_TC_	gtacatgagtctgagttccttg	322	58.21
R_TC_	atctggctattctccttagttg		56.35
F_TE_	cgaatactcttccgtcgatgtct	359	60.17
R_TE_	aggagctactgctgcacaactaa		60.17
F_TA35_	cagcgtgattcttaccagatatt	307	56.60
R_TA35_	tgttgcgagcattactgcgttta		58.39
F_TB_	gttgacttcactgattgtcgcacta	406	60.34
R_TB_	cgagcctgttaccttaaacttgg		60.17
F_TA66_	atatacctacttcgtcactgcc	352	58.21
R_TA66_	tttccttgttcatctattccac		54.48

### Genetic stability of MVTT_EAB_

BHK-21 cells were infected with MVTT_EAB_ and serially passaged 20 times to detect any reverse mutations of the deleted fragments (Guirakhoo et al., 1999). The amplification protocol consisted of 95°C for 5 min; 35 cycles of 95°C for 30 s, 57°C for 30 s, and 72°C for 30 s; and a final extension at 72°C for 10 min. The VVTT genome was used as a control to determine the genetic stability of MVTT_EAB_. The sequences of the primers used for identification are shown in Table 1.

### Growth curve

The BHK-21, HeLa, PK-15, MDCK, and Vero cells were cultured at a density of 5 × 10^5^ cells/well in six-well plates and then infected with MVTT_EAB_ or VVTT at 0.5 MOI. The cells were collected at 3, 12, 24, and 48 h after infection, and the viral titer in the BHK-21 cells was determined after three freeze-thaw cycles (Embry et al., 2011). The number of plaque-forming units (PFU) contained in 1 mL of viral fluid was calculated as follows: PFU/mL = (number of viral plaques × dilution ratio)/inoculation volume.

### MTS assay

The BHK-21, HeLa, PK-15, MDCK, and Vero cells were cultured at a density of 1 × 10^4^ cells/well in 96-well plates for 24 h at 37°C in a 5% CO_2_ atmosphere. Then the cells were infected with MVTT_EAB_ or VVTT at 0.5 MOI/well, and the infected cells were cultured at 37°C with 5% CO_2_. At 24, 48, 72, and 96 h, 20 mL of MTS solution (Promega) was added to each of the 96-well plate, which was cultured for 1 h at 37°C in a 5% CO_2_ atmosphere. Subsequently, we measured the absorption values at a wavelength of 490 nm using a microplate reader, which indirectly reflected the number of viable cells. Cell viability was calculated according to the following formula: 100 × (absorbance of culture in the treated wells/absorbance of culture in the control wells) (Mosmann, 1983; Li et al., 2006).

### Weight changes in mice after infection with MVTT_EAB_

Five-week-old BALB/c mice were inoculated intranasally with 1 × 10^5^ PFU/20 μL PBS, 1 × 10^6^ PFU/20 μL PBS, or 1 × 10^7^ PFU/20 μL PBS of MVTT_EAB_ or VVTT; a control group of mice was inoculated with PBS. There were 10 mice in each of the seven groups. The body weight of each mouse was recorded daily for 25 days after the inoculation (Vijaysri et al., 2008).

### Skin pathogenicity of MVTT_EAB_ in rabbits

In the rabbit skin pathogenicity assay, 1 × 10^6^ PFU/0.1 mL PBS, 1 × 10^7^ PFU/0.1 mL PBS, or 1 × 10^8^ PFU/0.1 mL PBS of MVTT_EAB_ or VVTT was injected intradermally into the backs of New Zealand white rabbits after their hair was shaved. Each concentration was injected into three rabbits. After the inoculation, skin lesions were formed on the back of the rabbits, and the lesions were measured with a vernier caliper and observed for 18 consecutive days.

### Detection of neurotoxicity in mice after infection with MVTT_EAB_

Three-week-old BALB/C mice (*n* = 10) were inoculated intracranially with 10 μL of MVTT_EAB_ or VVTT diluted with sterile PBS at doses of 1 × 10^5^ PFU/10 μL PBS, 1 × 10^6^ PFU/10 μL PBS, or 1 × 10^7^ PFU/10 μL PBS; the control group of mice was inoculated with PBS. Deaths were observed and recorded for 14 days after the inoculation. The intracranial 50% lethal infectious dose (ICLD50) was calculated using the Reed and Muench method (Reed and Muench, 1938).

### *In Vivo* immunogenicity assay

Six-week-old BALB/c mice (*n* = 10) were inoculated intramuscularly with 0.1 mL of MVTT_EAB_ or VVTT diluted with sterile PBS at a dose of 1 × 10^6^ PFU/0.1 mL PBS, and the control group of mice was inoculated with PBS. The first immunization was performed after collection of the first blood sample, and blood samples were collected every week after the first immunization. Three weeks later, booster immunization via the same route and of the same dose was performed. At the end of the fifth week, the mice were euthanized after the blood samples were collected. All serum samples were separated from the mouse blood samples, and the serum level of IL-2, IL-4, IL-10, and IFN-γ was detected using ELISA kits (GBD). And neutralization assay was performed as described previously (Kan et al., 2012). The results were calculated using the method of Reed and Muench (Reed and Muench, 1938).

### Statistical analysis

Statistical analysis was conducted using data from at least three independent experiments. SPSS or SigmaStat 3.5 (Systat Software) was used for the analysis. *P* < 0.05 was considered to indicate statistical significance. Data are presented as the mean ± standard deviation (*SD*) values.

## Results

### Construction and screening of the recombinant virus MVTT_EAB_

The shuttle plasmids were identified by double-restriction enzyme digestion with *Eco*RI and *Pst*I. The EGFP fragment (720 bp) was obtained by double digestion of the shuttle vectors pTC-EGFP, pTE-EGFP, pTA35-EGFP, pTB-EGFP, and pTA66-EGFP. These results indicated that the five shuttle plasmids were constructed successfully (Figures 1A–C).

**Figure 2:**
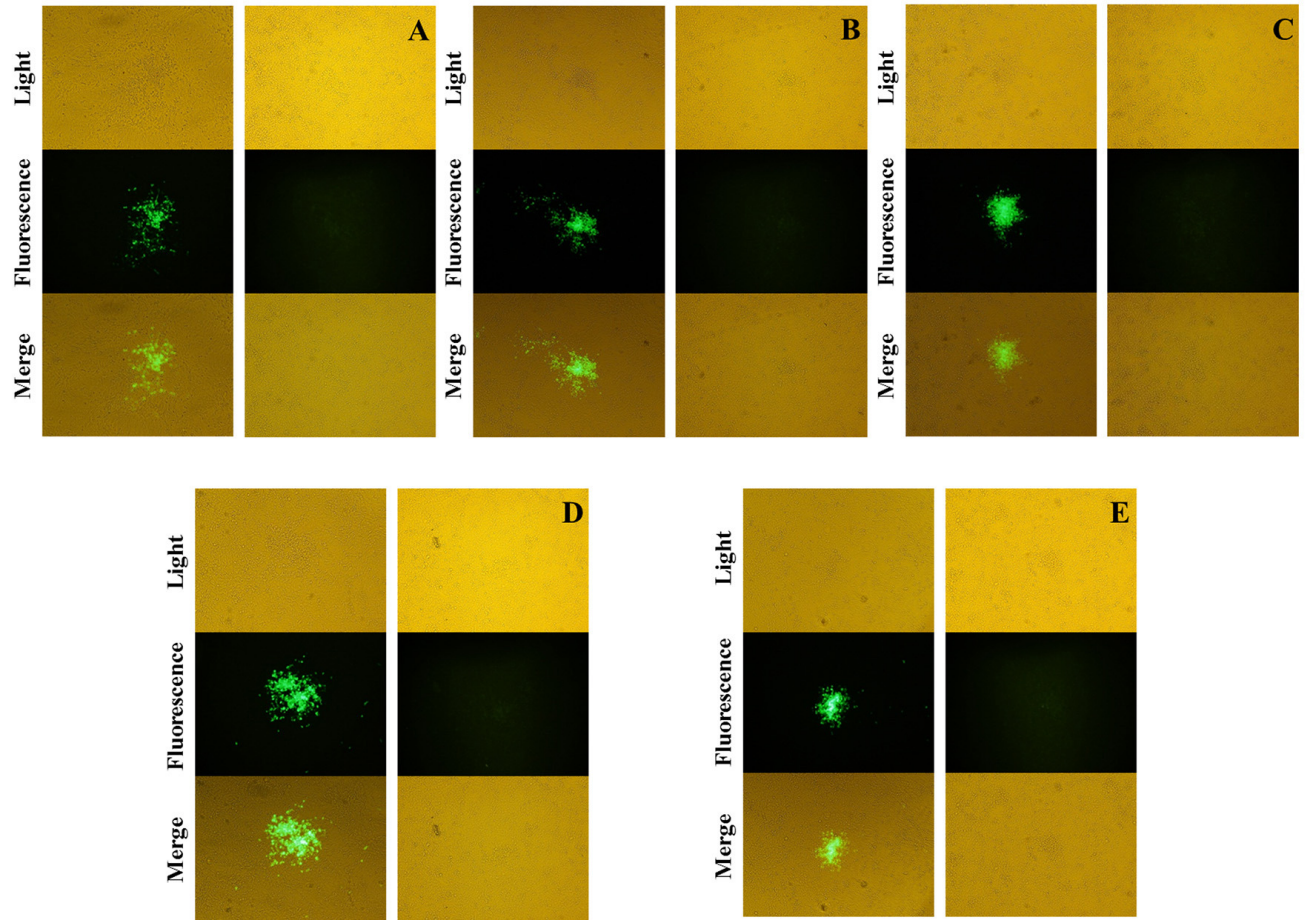
Screening and identification of mutants. The fused EGFP was expressed by the mutants rVVTT-C^−^EGFP^+^ **(A)**, rVVTT-C^−^E^−^EGFP^+^ **(B)**, rVVTT-C^−^E^−^A35^−^EGFP^+^ **(C)**, rVVTT-C^−^E^−^A35^−^B^−^EGFP^+^ **(D)**, and rVVTT-C^−^E^−^A35^−^B^−^A66^−^EGFP^+^ (MVTT_EAB_-EGFP^+^) **(E)** in BHK-21 cells. The virus-infected cells were identified by visualization of isolated fluorescent plaques in the same visual fields. Non-fluorescent plaques were observed for rVVTT-C^−^ **(A)**, rVVTT-C^−^E^−^ **(B)**, rVVTT-C^−^E^−^A35^−^ **(C)**, rVVTT-C^−^E^−^A35^−^B^−^ **(D)**, and rVVTT-C^−^E^−^A35^−^B^−^A66^−^ (MVTT_EAB_) **(E)** mutants in BHK-21 cells (magnification 200×, **A–E**).

The recombinant shuttle plasmid pTC-EGFP was co-infected/transfected into BHK-21 cells with VVTT, and the recombinant VV rVVTT-C^−^EGFP^+^ was obtained by 10 rounds of fluorescence plaque screening. The EGFP gene of rVVTT-C-EGFP^+^ was knocked out with the Cre-loxP system, and then the recombinant vaccinia virus rVVTT-C^−^ that did not contain the TC7L–TK2L genes was obtained by 10 rounds of fluorescence plaque screening (Figures 1, 2). The same screening method was used to knock out the other deletion fragments and construct a multi-gene-deleted attenuated strain of VVTT (MVTT_EAB_) in which five gene fragments and an exogenous selectable marker gene were deleted (Figure 2).

**Figure 3:**
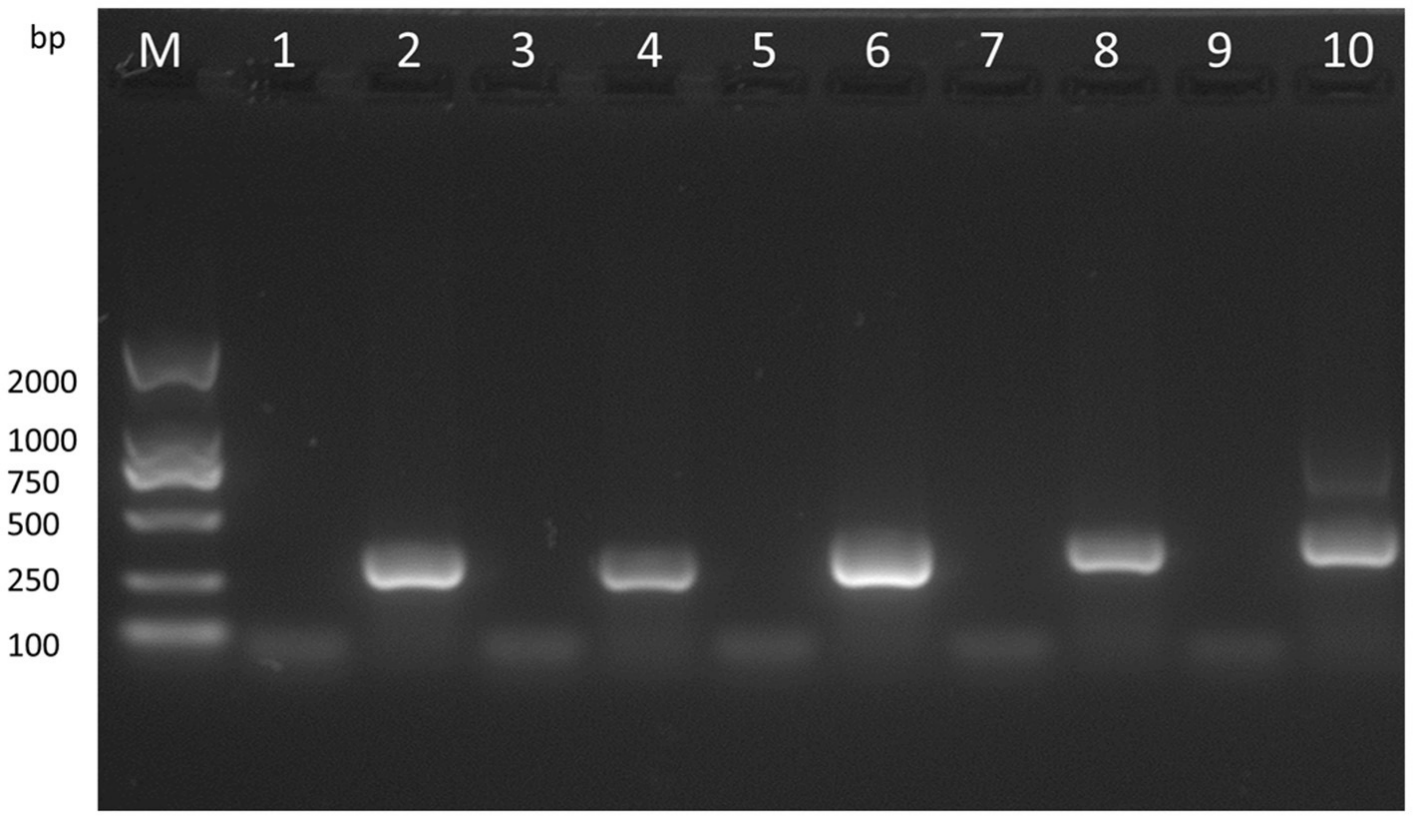
Analysis of the recombinant virus MVTT_EAB_ by PCR. PCR was performed to identify the final mutant MVTT_EAB_. Lane 2: positive control containing the TC7L-TK2L gene (322 bp), lane 4: positive control containing the TE3L gene (359 bp), lane 6: positive control containing the TA35L gene (307 bp), lane 8: positive control containing the TB13R gene (406 bp), lane 10: positive control containing the TA66R gene (352 bp), and lanes 1, 3, 5, 7, and 9: PCR products of the MVTT_EAB_ genome.

With the VVTT genome as the template, we obtained products of five different sizes by PCR amplification: TC, 322 bp; TE, 359 bp; TA35, 307 bp; TB, 406 bp; TA66, 352 bp (Figure 3). With the MVTT_EAB_ genome as the template, PCR amplification under the same conditions did not produce similar bands. Then, the virus was identified by sequencing, and the results showed that the five target gene fragments were knocked out from the VVTT genome: TC-TK, TE, TA35R, TB13R, and TA66R.

### Genetic stability of MVTT_EAB_

**Figure 4:**
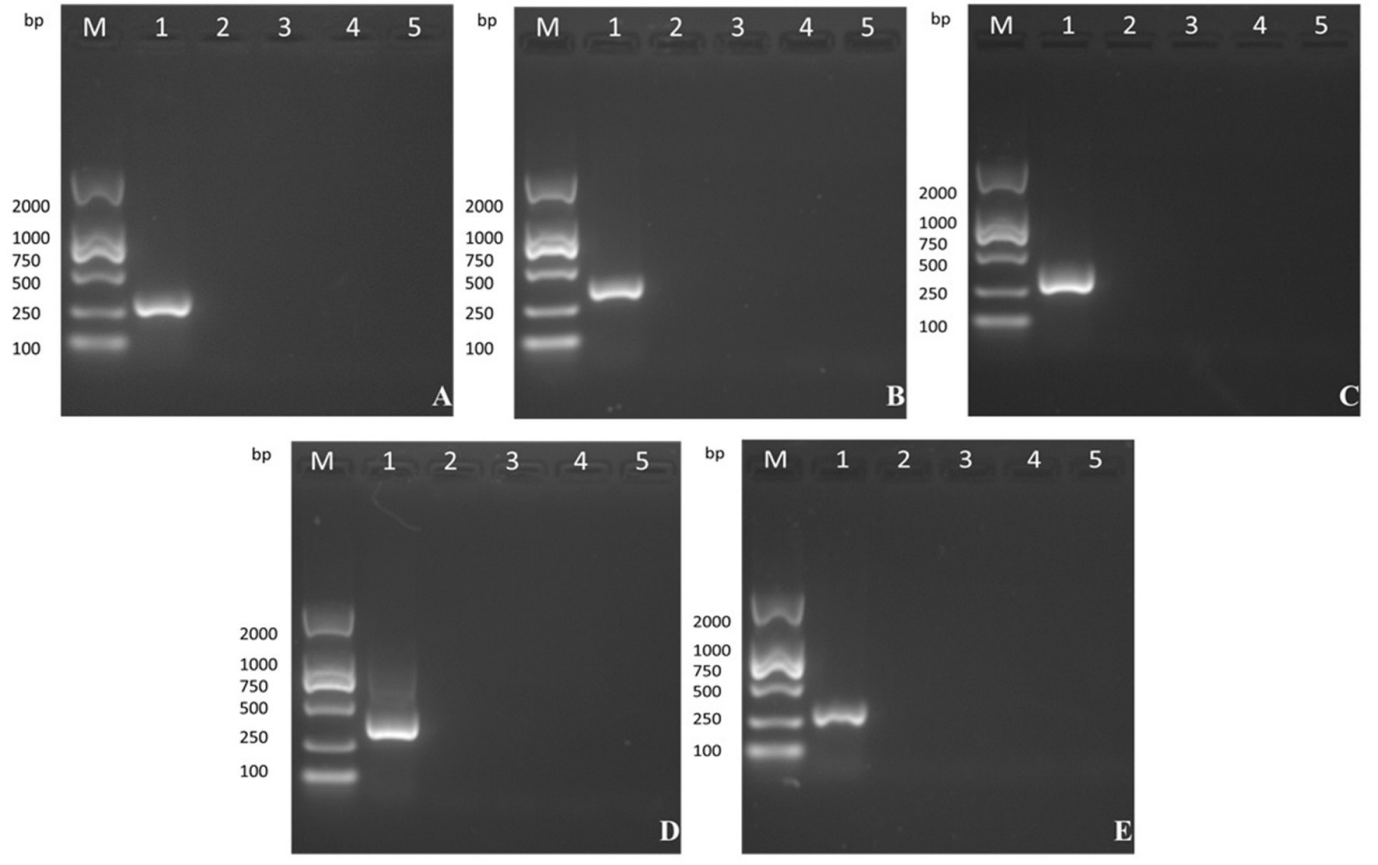
Evaluation of the genetic stability of the recombinant virus in BHK-21 cells after 5, 10, 15, and 20 passages. **(A)** TC7L-TK2L gene of MVTT_EAB_ (lanes 2–5), **(B)** TE3L gene of MVTT_EAB_ (lanes 2–5), **(C)** TA35L gene of MVTT_EAB_ (lane 2–5), **(D)** TB13R gene of MVTT_EAB_ (lanes 2–5), and **(E)** TA66R gene of MVTT_EAB_ (lanes 2–5). PCR of all gene-deleted mutants produced negative results, compared to the corresponding positive PCR controls (lane 1).

The five products of different sizes that were obtained by PCR amplification of the VVTT genome corresponded to the deleted fragments in the recombinant virus MVTT_EAB_: TC, 322 bp; TE, 359 bp; TA35, 307 bp; TB, 406 bp; TA66, 352 bp (Figure 4). However, PCR amplification of the 5, 10, 15, and 20th generation of the MVTT_EAB_ genome under the same conditions did not produce the corresponding bands. These results showed that MVTT_EAB_ had good genetic stability.

### Replication of MVTT_EAB_ in cells

**Figure 5:**
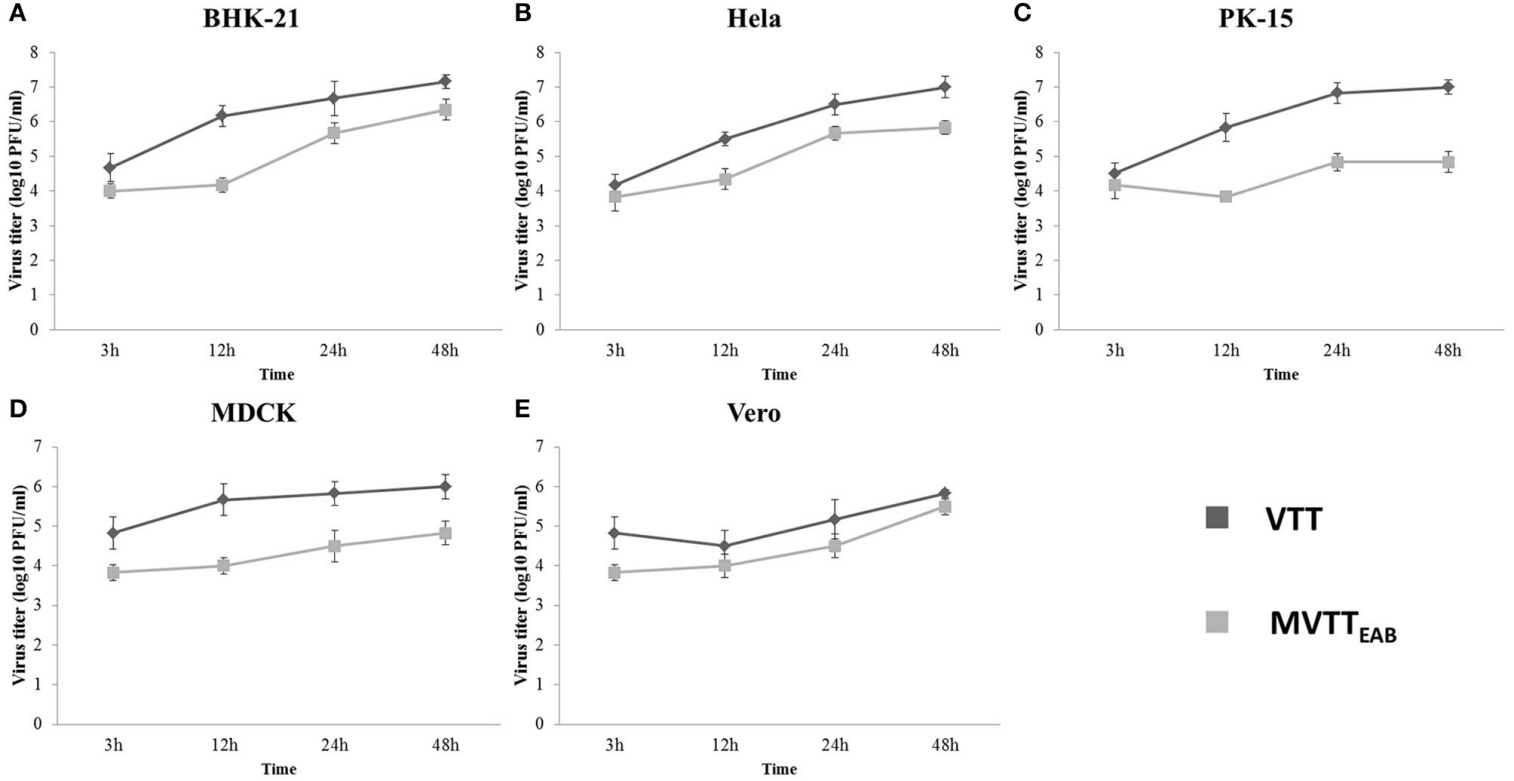
*In vitro* replication of MVTT_EAB_ and VVTT. Growth curve of MVTT_EAB_ and VVTT in BHK-21 **(A)**, HeLa **(B)**, PK-15 **(C)**, MDCK **(D)**, and Vero **(E)** cells. Cells were infected with MVTT_EAB_ and VVTT at an MOI of 0.5, and the virus titers were measured at 3, 12, 24, and 48 h after infection.

As shown in Figure 5, MVTT_EAB_ and VVTT showed similar growth trends in the same cell lines, but the viral titer decreased. The results indicated that the deletion of the gene segments in MVTT_EAB_ did not influence normal replication of the virus in the cells.

### Cell virulence of MVTT_EAB_

**Figure 6:**
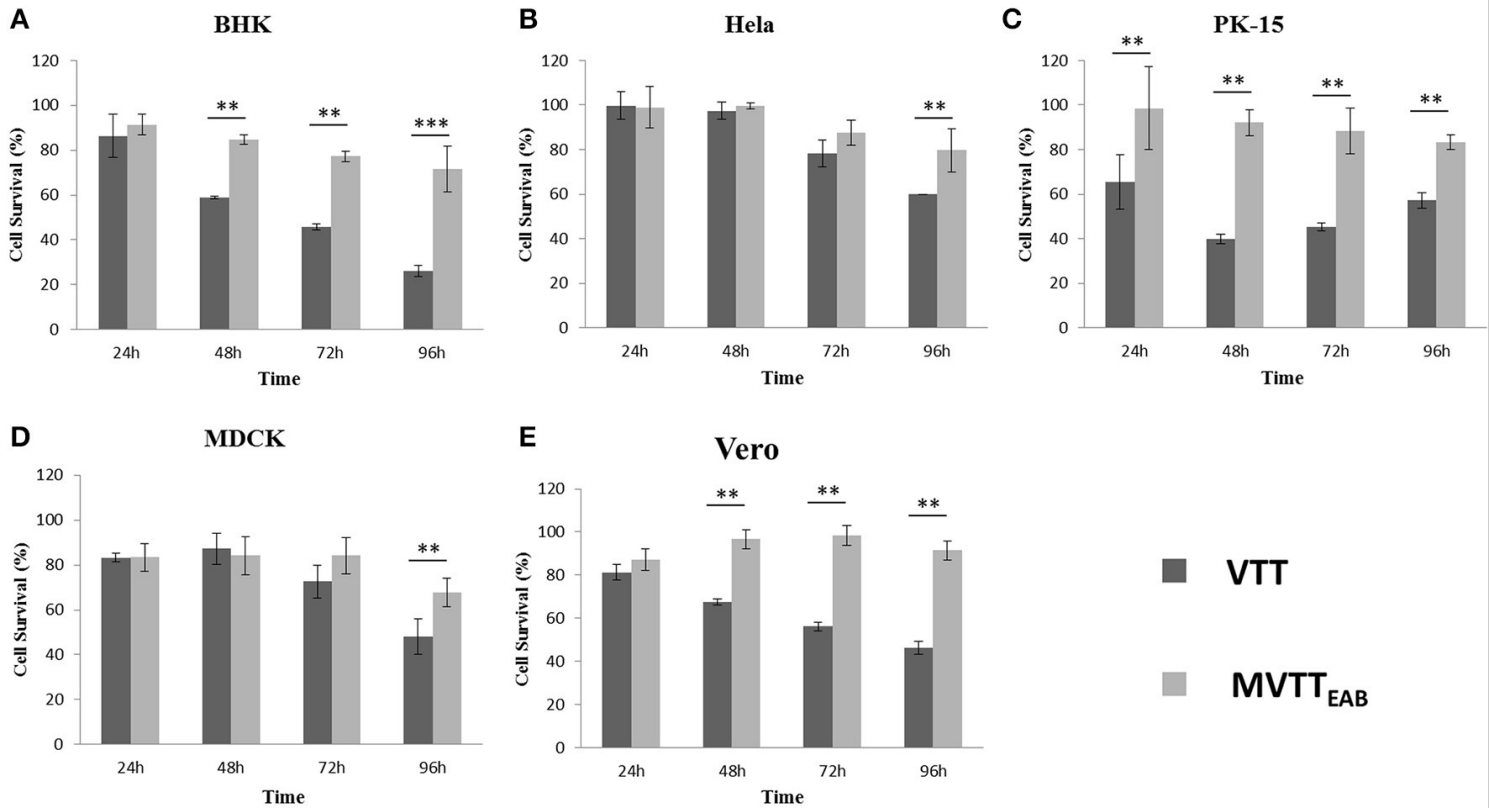
*In vitro* cytotoxicity of MVTT_EAB_ and VVTT. BHK-21 **(A)**, HeLa **(B)**, PK-15 **(C)**, MDCK **(D)**, and Vero **(E)** cells were seeded in 96-well plates (1 × 10^4^ cells/well) and infected with MVTT_EAB_ and VVTT at an MOI of 0.5. Cell viability was measured every 24 h over a 96-h period. All measurements were performed in triplicate. Data are presented as the mean ± standard deviation (*SD*) values. When *P* < 0.01 (^**^) or *P* < 0.001 (^***^), the difference was considered to be significant.

BHK-21, HeLa, PK-15, MDCK, and Vero cells are sensitive to VVTT, which easily replicates in these cells. Here, the cytopathic effects of the recombinant virus MVTT_EAB_ were detected and analyzed through MVTT_EAB_ and VVTT infection of the five cell lines (Figures 6A–E). As shown in Figure 6, the five cell types that were infected with VVTT exhibited varying levels of cytocidal effects. The number of living cells decreased with time, and in the BHK-21, PK-15, and Vero cells, the cell viability rate was 30, 60, and 50%, respectively, at 96 h. The cytopathic effect of MVTT_EAB_ on the growth of cells was lower than that of VVTT. From the data in Figure 6, it can be seen that in the BHK, PK-15, and Vero cells, the cell survival rate of the VVTT-infected cells was significantly lower than that of the MVTT-infected cells at 48 and 72 h (*P* < 0.01). At 96 h, the cell survival rate of the VVTT-infected cells (of all five cell types) was significantly lower than that of the MVTT-infected cells at 48 and 72 h. The difference was more significant in the BHK cells than in the other cell types (*P* < 0.001). The results indicated that the absence of the removed gene segments resulted in a decrease in the virulence of MVTT_EAB_. This demonstrated that the recombinant virus would be a safer vector or vaccine for vaccination than VVTT.

### Lesion formation in rabbits infected intradermally with MVTT_EAB_

**Figure 7:**
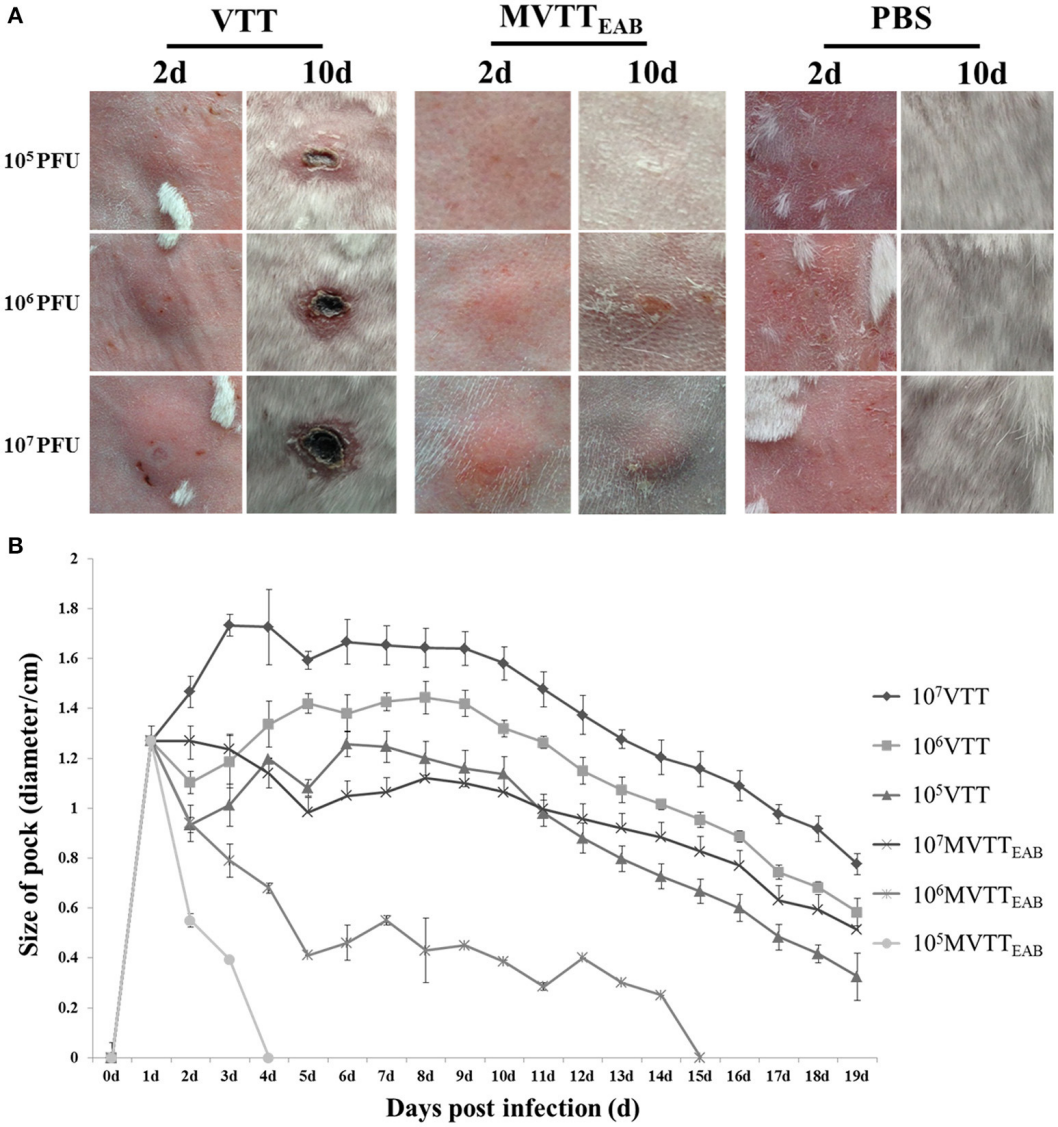
Virulence of MVTT_EAB_ or VVTT in rabbits. Rabbits were infected with 10^5^, 10^6^, or 10^7^ PFU of MVTT_EAB_ or VVTT (as a positive control) or PBS (as a negative control) by intradermal injection. Infection with 10^5^, 10^6^, and 10^7^ PFU of MVTT_EAB_ resulted in mild swelling by day 2 that subsided by day 4, and no visible scars were observed by day 10 at the sites of infection **(A)**. By contrast, infection with VVTT resulted in the formation of relatively severe lesions at the infection site with ulcerations; the size of the lesions increased until they peaked on day 2, after which they gradually decreased but left scars **(A)**. The diameter of the lesions was associated with the VVTT and MVTT_EAB_ dose **(B)**.

MVTT_EAB_ and wild-type VVTT were inoculated intradermally into the back of the rabbits, and the size of the pock lesions formed was measured daily for 18 days. The pock lesions induced by VVTT were extremely obvious, while the lesions induced by MVTT_EAB_ were much smaller. Furthermore, the higher viral titers of both VVTT and MVTT_EAB_ resulted in the formation of larger pock lesions. In addition, the trends in the size of the pock lesions formed by the two virus infections over time are similar: that is, the size of the pock lesions first increased and then decreased. As shown in Figure 7A, the pock lesions induced by VVTT became red and swollen from the next day of the inoculation. The size of the VVTT-induced lesions peaked on day 2, after which they gradually started festering. This was followed by scab formation, and on the tenth day, an obvious scab could be observed. At the end of the experiment, the size of the pock lesions induced by VVTT had been reduced, but they had not all disappeared. In contrast to the VVTT-induced lesions, the pock lesions produced by MVTT_EAB_ infection only appeared as a slight swelling on day 2, which subsided on day 4 for the lowest MVTT_EAB_ titer. From the data in Figure 7B, it can be seen that the size of pock lesions formed by MVTT_EAB_ infection at the lowest and medium viral titer were significantly smaller than that of lesions formed by VVTT infection at the lowest, medium and highest titer (*P* < 0.05). No significant difference was observed between the size of the pock lesions formed by MVTT_EAB_ infection at the highest viral titer and the MVTT infection at the lowest titer, but the former did not result in the formation of a fester or scab (*P* > 0.05). Thus, the deletion of the five genes in MVTT_EAB_ significantly reduced its virulence compared to wild-type VVTT. The dose-dependent pattern observed was comparable to that of VVTT and WR previously reported in mice (Brandt and Jacobs, 2001; Fang et al., 2005).

### MVTT_EAB_ virulence in BALB/c mice

**Figure 8:**
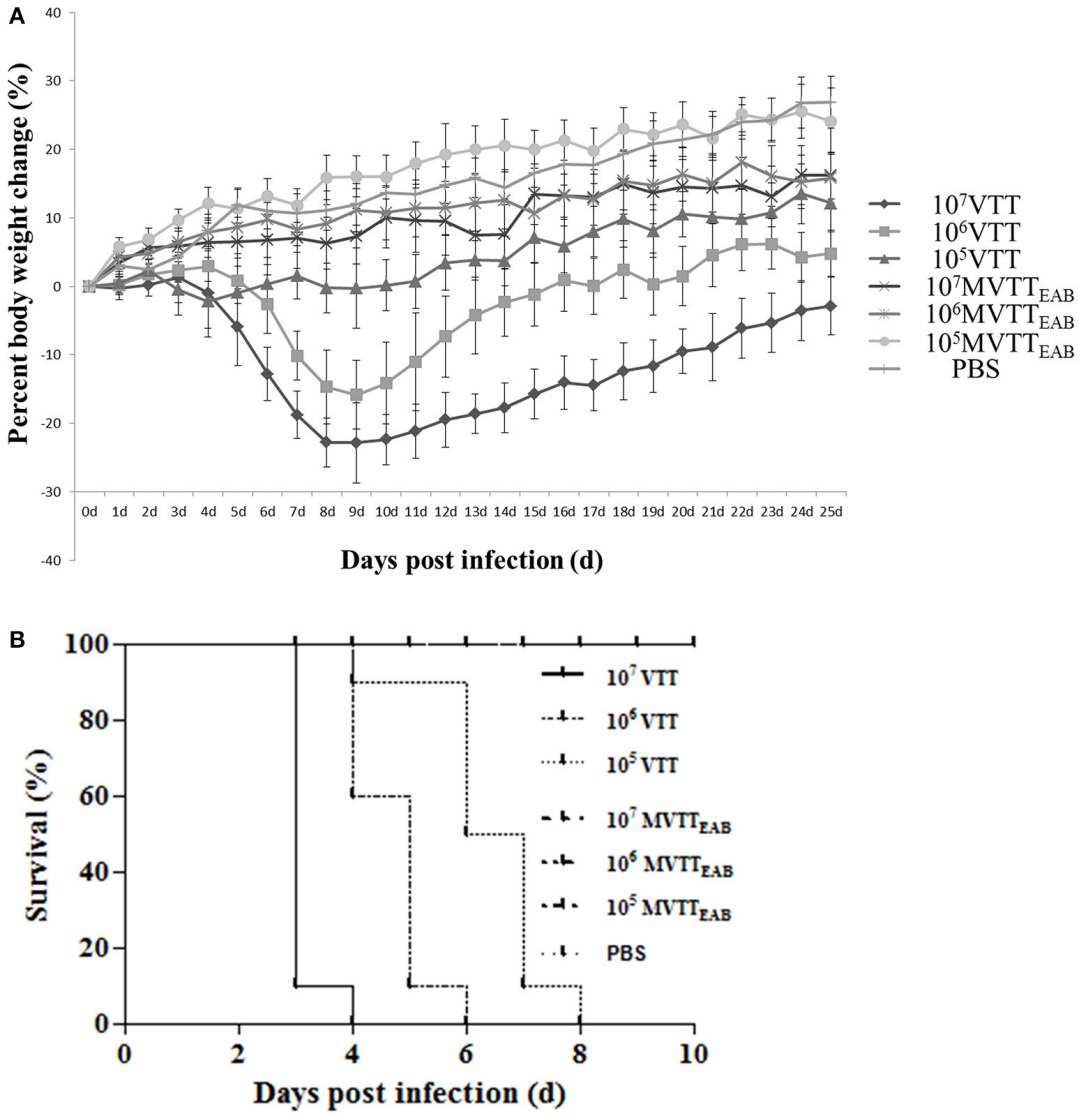
Virulence of VVTT and MVTT_EAB_ in mice after intranasal and intracranial infection. **(A)** Body weight changes were monitored in mice that were intranasally infected with 10^5^, 10^6^, or 10^7^ PFU of MVTT_EAB_ or VVTT (as a positive control) or PBS (as a negative control). Error bars indicate the standard error of the mean, and differences between groups were determined by two-way repeated measures analysis of variance. Mice inoculated with VVTT showed significant signs of illness, and a clear positive correlation was found between the viral dose and weight loss (*P* < 0.05). No distinct weight changes or signs of illness were observed in the animals inoculated with MVTT_EAB_ or PBS. **(B)** Mice were inoculated intracranially with 10^5^, 10^6^, or 10^7^ PFU of MVTT_EAB_ or VVTT (PBS in the negative control group), and the survival rates were observed for 14 days. All the mice inoculated with MVTT_EAB_ survived, while all the mice infected with VVTT died.

The recombinant virus MVTT_EAB_ and wild-type VVTT were intranasally inoculated into 5-week-old female BALB/c mice, and their body weights were observed for 25 days. As shown in Figure 8A, the weight of the mice infected with the highest dose of VVTT decreased from the fourth day after inoculation and reached the lowest level on the ninth day, with an average reduction of 22.8%. The weight started to gradually increase on the tenth day, but until the end of the experiment, the mean body weight of the VVTT group was significantly lower than that of the MVTT_EAB_ and PBS control groups (*P* < 0.05). The change in body weight in the low- and middle-dose VVTT group was similar to that in the high-dose VVTT group, but the degree of weight loss and period over which the weight were significantly different (*P* < 0.05). The weight of the mice in all the three MVTT_EAB_ groups did not decrease, and the trend in body weight changes was similar to that of the PBS control group. Further, the low-dose MVTT_EAB_ group showed more obvious weight gain than the other two groups with higher MVTT_EAB_ doses (*P* < 0.05). The results showed that the loss of body weight was positively correlated with the viral titer of the inoculated mice, and that the virulence of MVTT_EAB_ was lower than that of VVTT. These findings indicate that the deletion of the five genes in MVTT_EAB_ significantly reduced the *in vivo* virulence of VVTT.

The recombinant VV MVTT_EAB_ and wild-type VVTT were intracranially inoculated in mice for 14 days. Mice infected with VVTT exhibited neurological symptoms such as scruffy fur, lassitude, convulsions, and stiffness; the mice in the VVTT groups finally died. By comparison, mice infected with the three doses of MVTT_EAB_ did not exhibit such symptoms and were alive at the end of the observation period. Survival curves drawn for the mice (Figure 8B) showed that mice inoculated with the three different doses of MVTT_EAB_ showed 100% survival until the end of the experiment (Figure 8B), which was significantly different from the survival rate of the VVTT-infected mice (*P* < 0.05). On the fourth day after inoculation with 1.0 × 10^7^ PFU of VVTT, the survival rate was 0%, and on the sixth and eighth day after inoculation with 1.0 × 10^6^ PFU and 1.0 × 10^5^ PFU of VVTT, the survival rate was 0%. The ICLD_50_ of VVTT-inoculated mice was 1.16 × 10^4^ PFU. The results showed that the deletion of the five genes in MVTT_EAB_ significantly reduced the neurotoxicity of the virus in mice. In short, the safety of the recombinant vaccinia virus in mice was improved by knocking out multiple genes, which improved its potential as a vaccine vector or vaccine for MVTT_EAB_.

### Humoral and cellular immune response to infection with MVTT_EAB_

**Figure 9:**
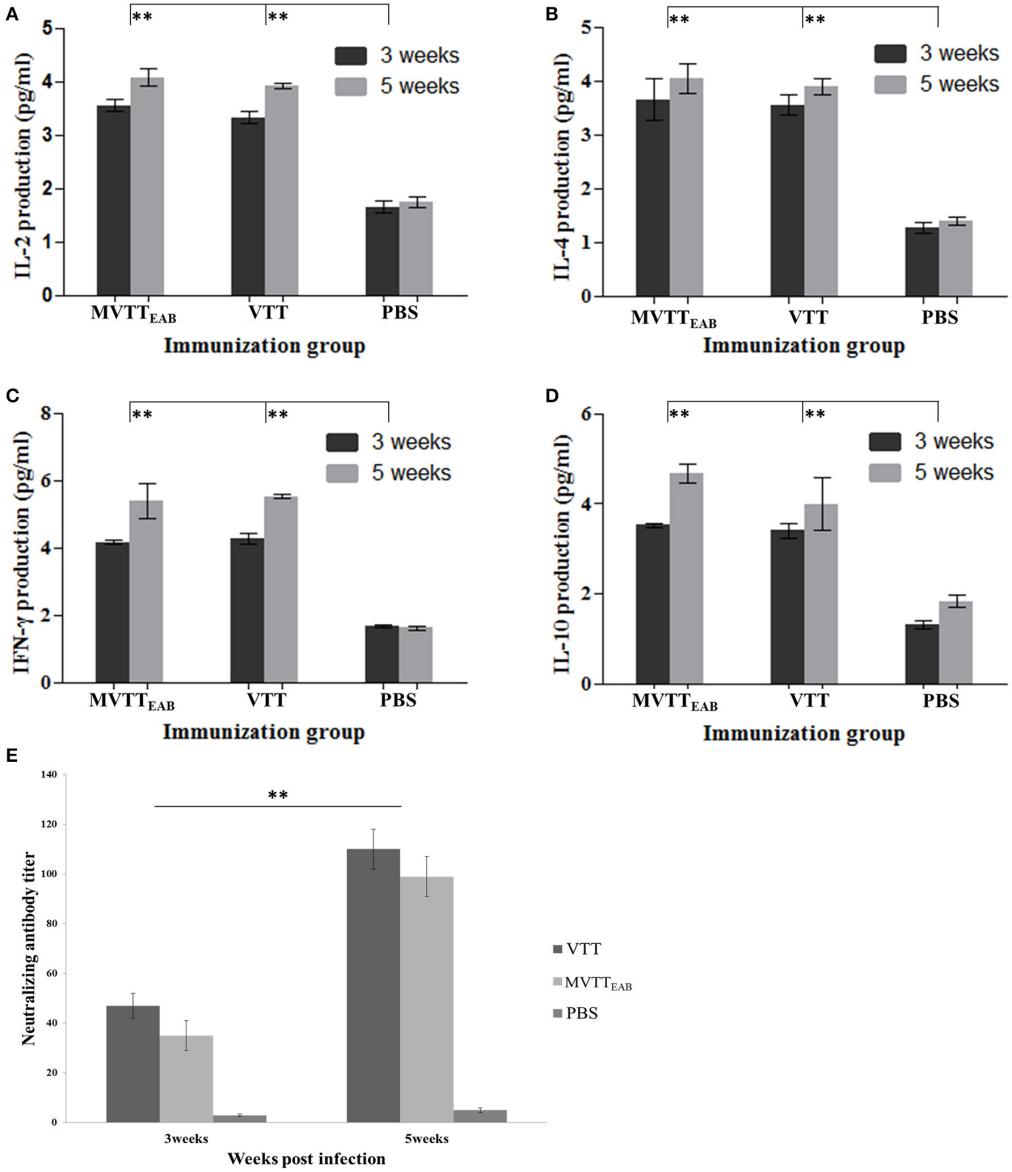
Immune responses to MVTT_EAB_ or VVTT in vaccinated mice. BABL/c mice were inoculated intramuscularly with 10^6^ PFU MVTT_EAB_ or VVTT, and given a booster immunization of the same dose in 0.1 ml PBS 3 weeks later. The serum samples were harvested at weeks 3 and 5 after immunization. The IL-2 **(A)**, IL-4 **(B)**, IFN-γ **(C)**, and IL-10 **(D)** levels in the two vaccinated groups were measured by mouse ELISA kits. All measurements were performed in triplicate. Data are presented as the mean ± *SD* values. The neutralization antibody titer was calculated by determining the highest serum dilution required to generate a 50% viral plaque reduction **(E)**. All measurements were performed in triplicate. Data are presented as the mean ± *SD* values. When *P* < 0.01 (^**^), the difference was considered to be significant.

The serum levels of IL-2, IL-4, IL-10, and IFN-γ were measured in all the mouse groups with mouse serum cytokine assay kits at the third and fifth weeks after immunization. The results indicated that the levels of IL-2, IL-4, IL-10, and IFN-γ in mouse serum in the VVTT- and MVTT_EAB_-infected groups were significantly higher than those in the PBS control group after the first immunization and the booster immunization (Figures 9A–D) (*P* < 0.01). In contrast, no significant differences were observed between the MVTT_EAB_-infected groups and VVTT-infected groups (*P* > 0.05). The results indicated that despite the deletion of the five genes in MVTT_EAB_, the virus could still induce high levels of these cytokines and maintain its immunogenicity as a vaccine.

The mice were immunized with MVTT_EAB_ and VVTT at a dose of 1 × 10^6^ PFU to detect neutralizing antibodies in the serum. As shown in Figure 9E, the serum neutralizing antibody titer of the MVTT_EAB_ and VVTT groups was not significantly different after immunization (*P* > 0.05). After the booster immunization, the serum neutralizing antibody titers of the MVTT_EAB_ and VVTT groups increased by about five times and reached about 100. Thus, the recombinant MVTT_EAB_ virus retains its immunogenicity as a vaccine despite the deletion of five genes and can stimulate a strong systemic immune response.

## Discussion

In this study, in order to construct a safer and effective attenuated VVTT strain, the genome of this strain was modified on the basis of analysis of the whole genome of several VV strains that are widely used. The non-essential gene fragments of the viral genome were deleted using the homologous recombination technique and the Cre/loxP system. The deleted gene segments were TC7L-TK2L, TE3L, TA35R, TB13R, and TA66R. TC7L-TK2L is not only a host-range gene but also a host defense regulatory gene (McFadden, 2005; Zhu et al., 2007; Yu et al., 2010). TE3L is a virulence-associated gene that inhibits the antiviral activity of interferon, and it is also associated with host range determinants, host defense regulation factors, and cell apoptosis regulatory factors (Wang et al., 2012). TA35R is a vaccinia A-type inclusion body protein gene fragment that is homologous with A26L of Vaccinia virus Copenhagen strain-encoded A-type envelope proteins (Rehm and Roper, 2011). TB13R is an immunoregulatory gene that encodes serine protein inhibitor and is also involved in the Fas-mediated death receptor pathway and lipoxygenase pathway. TA66R is a gene encoding viral hemagglutinin, which has the same function as the A56R gene of the Vaccinia virus WR strain and plays a role in inhibiting cell fusion (Buller et al., 1985). The absence of these genes in the newly constructed MVTT_EAB_ strain was confirmed by PCR. Additionally, the genetic stability of the newly constructed MVTT_EAB_ strain was confirmed, and it was also shown that the deletion of these segments did not affect the replication ability of the virus.

In the present study, a significant decline in the virulence of MVTT_EAB_ was verified in both *in vitro* and *in vivo* experiments. Similar to our findings, other studies have also reported that the deletion of certain non-essential gene fragments led to a decrease in virulence in VV strains. For example, it has been shown that the virulence of VV is reduced after deletion of A35R but does not affect the size of the plaque formed; thus, A35R is a non-essential gene for viral replication that is associated with the virulence of VV (Roper, 2006). Further, deletion of C, C2L, and N1L has also been found to result in a significant reduction in the virulence of VV (Legrand et al., 2004). Another study showed that the virulence decreased significantly in the absence of B13L in the WR strain in nude mice and normal mice, while humoral immunity and cellular immune response were still high (Legrand et al., 2004). Knockout of E3L has also been shown to inhibit interferon activation and antiviral response (Langland and Jacobs, 2002), which means that deletion of this gene attenuates viral virulence (Wang et al., 2012). Altogether, the present findings as well the findings of previous studies show that the deletion of these specific non-essential genes that are not associated with viral replication can attenuate the virulence of VV and therefore improve its prospects as a vaccine.

In our *in vitro* experiments, the MTS assay was used to detect the cytotoxicity of the recombinant virus MVTT_EAB_ that was constructed and wild-type VVTT in five different cell lines. From 48 h after infection, the survival rate of the infected cells began to significantly differ between the VVTT- and MVTT_EAB_-infected groups, with the survival rate of the cells infected with MVTT_EAB_ being significantly higher than that of the cells infected with VVTT. Thus, the cytotoxicity of VVTT was significantly weakened as a result of deletion of the gene fragments. The recombinant virus MVTT_EAB_ therefore seems to have better safety than the wild-type VVTT, which means that it may be safer for use as a viral vector or vaccine for disease prevention/treatment.

In the skin lesion formation experiment in rabbits, the lesion size peaked on the third day after inoculation with VVTT, and then decreased slowly. On the contrary, only mild swelling was observed in the MVTT_EAB_ groups, and the swelling subsided in the low-dose group on the fourth day. Thus, deletion of the five gene fragments seems to have significantly reduced the skin damage caused by the wild-type VV in rabbits. Similarly, it has been shown that after inoculation of MVA, a recombinant VVTT strain, in rabbits, the lesions formed recovered at 12 days (Melamed et al., 2013).

In our *in vivo* virulence experiments in mice, the body weight of the mice was the lowest on the ninth day in the high-dose VVTT group, with an average reduction of 22.8%. In contrast, the weight trend of mice in the MVTT_EAB_ group was similar to that in the PBS control group. Thus, there was a significant reduction in the virulence of VV after deletion of the five gene fragments. Similarly, it has been reported that inoculation of MVA did not result in a decrease in the weight of the inoculated mice compared to mice inoculated with the wild-type virus (Melamed et al., 2013). With regard to its neurotoxicity, MVTT_EAB_ was found to be highly safe in BALB/c mice, as all the mice that were intracranially inoculated with MVTT_EAB_ survived with no neurological symptoms. This was in contrast to the observations in the VVTT-inoculated group, in which all the mice died on the eighth day after inoculation. The findings for MVTT_EAB_ are similar to those reported for MVA and NYVAC, which are the most commonly used attenuated VV strains in which multiple host-range genes, virulence genes and other non-essential genes are deleted (Tartaglia et al., 1992; Melamed et al., 2013).

Cytokines play an important role in the body's immune response, such as antiviral and mediated inflammatory responses. Research has shown that IL-4, IL-10, and IFN-γ play an important role in the immune response of mice to VV infection (van Den Broek et al., 2000). Most studies on the deletion of immunoregulatory genes in VV have shown that the absence of several VV genes can reduce the toxicity of the virus (Smith et al., 2013), but the effect on the immunogenicity of the virus is variable. For example, deletion of these immunomodulatory genes from different strains (mainly WR and MVA) was found to increase the immunogenicity of this virus: E3L, B15R/B16R, A41L, B22R, C12L, and C6L. However, the absence of immunomodulatory genes, such as B8R, was found to have no effect on virulence or pathogenicity, while the deletion of genes such as N2L and C16L was found to have no effect on immunogenicity (Alcami and Smith, 1995; Fahy et al., 2008; Ferguson et al., 2013). In addition, deletion of C12L, A44L, A46R, or B7R in MVA did not significantly affect VACV-specific CD8 + T cell immunogenicity in BALB/c mice (Cottingham et al., 2008). Based on the studies described above, we deleted five gene fragments, including TC7L-TK2L, to detect and analyze changes in the immunogenicity of MVTT_EAB_. The results of this study show that the degree of immune response in mice infected with MVTT_EAB_ is almost equal to that of VTT infection, and there is no significant difference (*P* > 0.05). Further, MVTT_EAB_ seems to have attenuated virulence compared to VVTT, which makes it safer for clinical application. In summary, MVTT_EAB_ has demonstrated excellent safety and immunogenicity than the wild-type virus and has potential as a new virus vector and vaccine for the prevention or treatment of diseases caused by different pathogens.

## Author contributions

Conceived and designed the experiments: YL, XL, LS, and NJ. Performed the experiments: YL, YZ, SC, WL, XY, SL, and NJ. Analyzed the data: YL, XL, LS, and NJ. Contributed reagents/materials/analysis tools: YZ, SC, WL, XY, SL, PX, and JH. Wrote the paper: YL and NJ. All authors read and approved the final manuscript.

### Conflict of interest statement

The authors declare that the research was conducted in the absence of any commercial or financial relationships that could be construed as a potential conflict of interest. The reviewer MF and handling Editor declared their shared affiliation.
